# Precipitable Water Vapour Retrieval from GPS Precise Point Positioning and NCEP CFSv2 Dataset during Typhoon Events

**DOI:** 10.3390/s18113831

**Published:** 2018-11-08

**Authors:** Xu Tang, Craig Matthew Hancock, Zhiyong Xiang, Yang Kong, Huib de Ligt, Hongkai Shi, Jonathan Arthur Quaye-Ballard

**Affiliations:** 1 Department of Civil Engineering, University of Nottingham Ningbo China, 199 Taikang East Road, Ningbo 315100, China; Craig.hancock@nottingham.edu.cn (C.M.H.); Huib.deligt@nottingham.edu.cn (H.d.L.); 2 The First Surveying and Mapping Institute of Zhejiang Province, Hangzhou 310012, China; zjsdychy@zjch.gov.cn; 3 Ningbo Meteorological Bureau, 118 Qixiang Road, Ningbo 315100, China; kong_yang1988@163.com; 4 School of Earth Science and Engineering, Hohai University, 8 Fochengxi Road, Nanjing 211100, China; shkcehui@hotmail.com; 5 Department of Geomatic Engineering, Kwame Nkrumah University of Science and Technology (KNUST), Kumasi +233, Ghana; quayeballard.soe@knust.edu.gh

**Keywords:** precise point positioning, NCEP CFSv2, CORS, precipitable water vapour changes, typhoon event

## Abstract

Radiosonde is extensively used for understanding meteorological parameters in the vertical direction. Four typhoon events, including three landfalls (MERANTI, NEPARTAK, and MEGI) and one non-landfall (MALAKAS), were chosen in analysing the precipitable water vapour (PWV) characteristics in this study. The spatial distribution of the three radiosonde stations in Zhejiang province does not meet the requirement in analysing changes in PWV during typhoon event. Global position system (GPS) observations are an alternative method for deriving the PWV. This enables improvements in the temporal–spatial resolution of PWV computed by the radiosonde measurements. The National Centers for Environmental Prediction (NCEP) re-analysed data were employed for interpolating temperature and atmosphere pressure at the GPS antennas height. The PWV computed from GPS observations and NCEP re-analysed data were then compared with the true PWV. The maximum difference of radiosonde and GPS PWV was not more than 30 mm at Taiz station. The Root-Mean-Square (RMS) of PWV differences between radiosonde and GPS was not more than 5 mm in January, February, March, November, and December. It was slightly greater than 5 mm in April. High RMS in May, June, July, August, September, and October implies that differences in GPS and radiosonde PWVs are evident in these months. Correlation coefficients of GPS and radiosonde PWVs were more than 0.9, indicating that the changes in GPS and radiosonde PWVs are similar. Radiosonde calculated PWVs were used for GPS PWV calibration for understanding the PWV changes during the period of a typhoon event. The results from three landfall typhoons show that the average PWV over Zhejiang province is increasing and approaching China mainland. In contrast, MALAKAS did not make landfall and shows a decreasing PWV trend, although it was heading to China mainland. Generally, the PWV change can be used to predict whether the typhoon will make landfall in these cases. PWV spatial distribution of MERANTI shows that PWV peaks change along the typhoon epicenter over Zhejiang province.

## 1. Introduction

**Figure 1:**
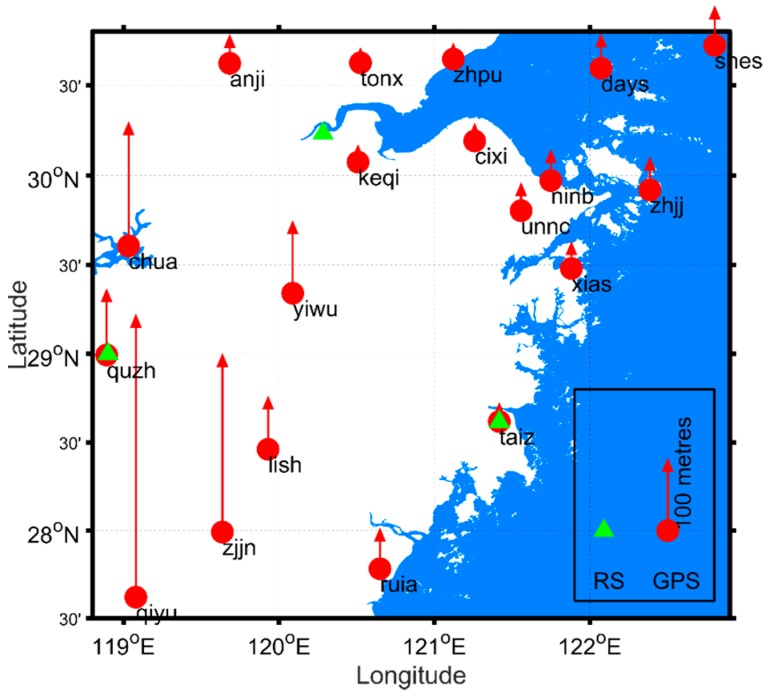
Location of continuously operating reference stations (CORS) (red dots), radiosonde stations (green triangle markers), and global navigation satellite system (GNSS) antenna WGS-84 geodetic ellipsoid height. GPS—global position system.

There are approximately 364 sensors with a coverage of 9400 km^2^ established in the City of Ningbo, Zhejiang, China. These sensors record hourly earth surface temperature, atmospheric pressure, wind speed and direction, humidity, and precipitation, among others. Meteorological measurement, distributed vertically, is an essential parameter for weather forecasting. The general approach in collecting vertical meteorological measurements is to use a radiosonde on a balloon travelling from the Earth’s surface to the stratosphere. The high resolution is required for understanding how atmospheric water vapour changes under extreme weather conditions (e.g., typhoon event). Radiosonde is extensively used in China for understanding meteorological parameters in the vertical direction. These radiosonde balloon stations are sparsely distributed in China. There are three radiosonde stations in Zhejiang province, where radiosonde balloons are launched manually twice a day at 07:15 and 19:15. These three stations cover an area of 105,500 km^2^ (Figure 1). In the case where strong winds blow the balloon to an unexpected route, spare balloons have to be launched and this comes with many challenges. Under extreme weather conditions, the meteorological parameters change within a short period. Therefore, launching radiosonde balloons twice a day cannot meet such requirements. Many typhoons pass through Zhejiang province every year, resulting in rainfall and downtown flooding. Real-time or near real-time water vapour detection helps to manage the urban infrastructure and reduce any cost as result of flooding. Densely distributed PWV detection stations will also help the local agency to forecast weather.

Global position system (GPS) signals are delayed while passing through the troposphere and ionosphere. Ionospheric delay can be estimated or significantly reduced by dual frequency carrier phase linear combination [1]. Tropospheric delay is known as zenith total delay (ZTD) or zenith path delay (ZPD) in some literature. ZTD can be precisely estimated by either global navigation satellite system (GNSS) double difference (DD) or precise point positioning (PPP) technology [2,3,4]. The GPS technique of ZTD estimation can improve the temporal resolution of PWV to 30 s or even less. The precise water vapour can be estimated in the near real-time, further than for the spatially resolved troposphere humidity field [5]. PPP estimation precision highly relies on the types of International GNSS Services (IGS) products, which are used for PPP data processing. The final version of IGS satellites products is recommended for computing precise ZTD using PPP. However, the products have latency of a few days, which limits the retrieval of GPS PWV for weather broadcasting. Research has revealed that PPP supports near real-time ZTD estimation if the real-time precise satellite clock and orbit products are available [6,7]. The IGS started to deliver real-time GNSS products data stream in 2013, which allows real-time PPP. Real-time satellite clock and orbit products were assessed for ZTD estimation using PPP [8]. Additionally, PPP convergence has to be mentioned for data processing. Tens of minutes have to be taken for the unknown parameters re-convergence and precise ZTD estimation, as a result of interruption of GPS data. PPP convergence performance is improved by using multi-GNSS [9]. In order to improve the current PPP estimation precision, the concept of PPP carrier phase integer property recovery was introduced [10,11]. ZTD with better accuracy can be achieved by real time PPP when the zero-difference carrier phase ambiguities are fixed [12]. PWV is important in numerical weather forecasting [13] and can be properly be segmented from ZTDs with the additional atmospheric pressure and temperature near GNSS antennas [14,15,16]. For instance, ground-based GPS and meteorology sensors were used in sensing the PWV during the Melbourne storm in 2010 [17]. Previous research has shown that PWVs retrieved from GPS were successfully used for short-term rainfall forecasting during a typhoon event [18,19]. The atmospheric water vapor changing event has been analyzed during extreme weather conditions [20,21]. PWV is determined using multiple parameters. However, previous research only considers one typhoon event at a time. A single typhoon event cannot represent the general regulation of PWV changes during the typhoon in a statistically significant manner. Therefore, this research assesses PWV during several typhoon events in Zhejiang during 2016.

Dense distribution of CORS (continuously operating reference stations) in Zhejiang province, China is originally deployed for precise positioning. Meteorological sensors are not equipped to the Zhejiang CORS. Re-analysed data are often used for interpolating the temperature and atmospheric pressure near the GNSS antenna for retrieving PWV [22,23,24]. Radiosonde data and the numerical weather prediction (NWP) model are other means of obtaining temperature and pressure information [25]. This is more precise than the re-analysed data and is normally used for weather broadcasting. Relief amplitude and temporal–spatial resolution re-analysed data have great influence on the accuracy of interpolated temperature and atmospheric pressure near the GNSS antenna. This study aims to analyse PWV errors and bias when the NCEP dataset is used for GPS meteorology. It further investigates the relief amplitude affecting GPS antennae for temperature and pressure interpolation using the NCEP dataset. PWV was calculated from raw readings of the radiosonde sensors. The results served as the true variable in comparison with the retrieval results from the GPS PPP and NCEP dataset. Finally, the retrieved PWVs were applied in understanding the changes in atmospheric water vapour during extreme weather conditions. Four typhoon events, including three landfalls (MERANTI, NEPARTAK, and MEGI) and one with no landfall (MALAKAS), were chosen in analysing the PWV characteristics in this study. The pathway of the non-landfall typhoon is in China and changed its direction by leaving China mainland when it had almost landfall. This approach was adopted because of the advantage of GPS PWV high spatial and temporal resolutions.

## 2. Methods

### 2.1. Data Description

GPS data gathered from the Zhejiang CORS were used for precise ZTD estimation. The time interval for the GPS observations in this study is 30 s and the cut-off angle is 10 degrees. The second version of Climate Forecast System (CFSv2) was made operational in March 2010 (https://rda.ucar.edu/datasets/ds094.0/#!description), and is available for providing up-to-date climate data [26]. Land surface temperature and atmospheric pressure at 0.5 degree horizontal resolution were used in interpolating those near the GPS antenna. These datasets are offered four times per day at 00:00, 06:00, 12:00, and 18:00 UTC time. Figure 1 shows the locations of GPS antennas distributed over Zhejiang province, between longitude 118.8° E and 122.9° E, and latitude 27.5° N and 30.8° N. These are also the locations of meteorological sensors and radiosonde balloon launch points. GPS antennae were mounted on the top of permanent pillars, which were not high above the land surface. Figure 1 also shows the antenna’s geodetic height above the surface of the WGS-84 ellipsoid. The stations located southwest of Zhejiang province have heights higher than those located in the northeast regions. Particularly, Qiyu station has a geodetic height of more than 400 m. This generally reveals the relief amplitude of Zhejiang province. The radiosonde station near Zhejiang CORS Taiz is named HOJA. The distance between Taiz and HOJA is 176.5 m. The radiosonde stations near Zhejiang CORS Quzh and Keqi stations are named QUZO and HAZH, respectively. The distance between the radiosonde and CORS stations at QUZO and HAZH is 1.14 km and 27.96 km, respectively. The radiosonde balloons record the atmospheric pressure, height, temperature, dew-point, and wind speed and direction of each layer from the Earth’s surface to atmospheric pressure of 100 hPa. All the readings from radiosondes balloons are used in understanding the vertical weather condition.

### 2.2. PWV Retrieved by GPS PPP and NCEP CFSv2 Re-Analysed Dataset

**Figure 2:**
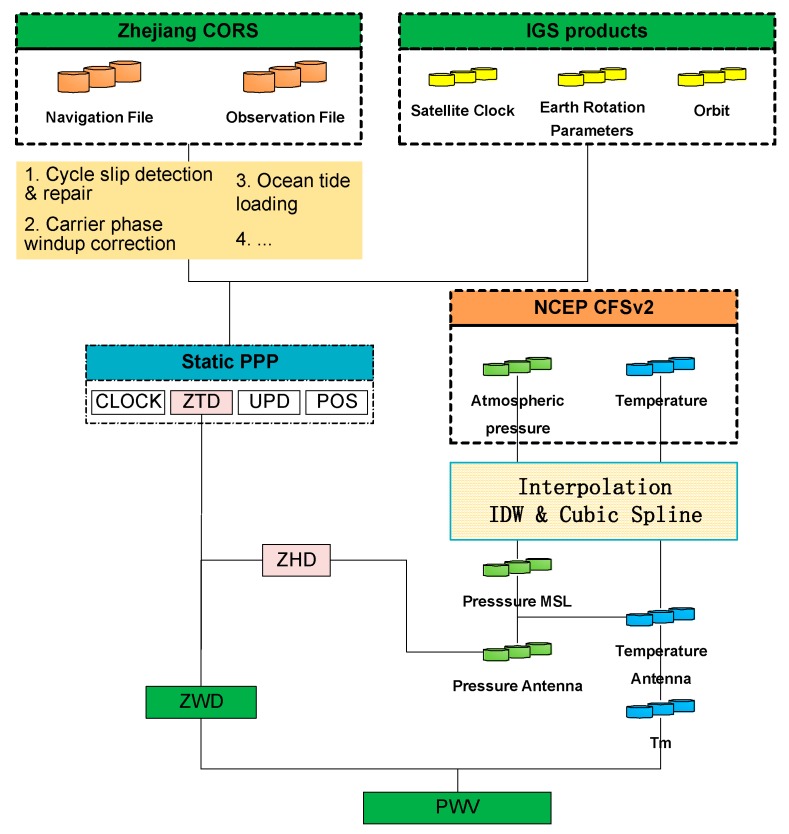
Flowchart for PWV retrieval from GPS PPP and NCEP sCFSv2 dataset. IGS—International GNSS Services; ZTD—zenith total delay; ZHD—zenith hydrostatic delay; ZWD—zenith wet delay; IDW—inverse distance weighted; UPD—uncalibrated phase delay; POS—position; MSL-mean sea level.

The GPS signal is delayed when propagating through the ionosphere and troposphere. The ionosphere-free (IF) linear combination removes the first order of ionospheric errors that makes up most of the total ionospheric delay. Assuming m satellites’ carrier phase observations at a specified epoch, the number of unknown parameters is 4+2×m, including three unknown position parameters, one receiver clock offset, m unknown float carrier phase ambiguity parameters, and m slant total delay. Various empirical mapping functions allow the slant total delay in the “line-of-sight” of each satellite to be expressed in the unique zenith direction, known as ZTD. This reduces the unknown troposphere delay parameters from m to 1. The most popular mapping functions are Neill Mapping Function (NMF) [27], Global Mapping Function (GMF) [28], and the Gridded Vienna Mapping Function 1 (VMF1) [29]. The GMF is designed with higher accuracy than NMF and it is most consistent with VMF1. Hence, GMF is chosen in this study. Using a mapping function, the number of unknown parameters is 5+m, of which only ZTD has to be estimated rather than the m slant total delays. Figure 2 shows the steps in retrieving PWV from the GPS and NCEP CFSv2 re-analysed dataset. The ZTD is first estimated by the RTKlib version 2.4.3 with the static positioning model. Cycle slip detection and repair, carrier phase windup correction, and ocean tide loading, among others, are required for the dataset preprocessing. Precise orbital and clock products, with 30 s and 5 s intervals, respectively, from center for orbit determination in Europe (CODE) were used in the PPP data processing strategy.

ZTD is divided into zenith hydrostatic delay (ZHD) and zenith wet delay (ZWD). ZHD is accurately determined by pressure. The Saastamoinen model can provide the ZHD with accuracy of 1 mm.

(1) $$\mathrm{ZHD}=\frac{0.0022768\times e_{s}}{1-0.00266\times \cos\left(2\varphi \right)-2.8\times 10^{-7}\times h}\;$$

In Equation (1), the unit of the ZHD is metres, $e_{s}$ is the atmospheric pressure at the antenna height with the unit hPa, φ is the latitude of antenna location, and h is the GPS station geodetic height in the unit of metre.

In this study, the temperature near the antenna is interpolated by the inverse distance weighted (IDW).

(2)  T¯=∑i=14(Tidi2)∑i=14(1di2), (i∈Z) 

where $d_{i}$ denotes the distance between the antenna and four grid notes, which are the nearer to the antenna location. $\bar{T}$ denotes the temperature near the GPS antenna. Only four temperature records can be interpolated in a day at each GPS station attributable to the NCEP CFSv2 re-analysed dataset, which is recorded at 6 h intervals. NCEP CFSv2 temperature and pressure are recorded with reference to the Earth’s surface and mean sea level, respectively. Cubic spline interpolation (CSI) is used for interpolating the temperature at a specified epoch. This was done at 30 s intervals in order to synchronize with the GPS data. In the same way, the atmospheric pressure ${\mathrm{Pres}}_{\mathrm{MSL}}$ can be interpolated by the IDW and CSI, respectively. The pressure offered by NCEP CFSv2 is at the mean sea level, which is different from temperature at the earth surface. The atmospheric pressure at the antenna height in Equation (1) can be calculated from ${\mathrm{Pres}}_{\mathrm{MSL}}$ in the equation below.

(3) $$\;e_{s}={\mathrm{Pres}}_{\mathrm{MSL}}\times {\left(1-\frac{0.0065\times h}{\bar{T}-0.0065\times h+273.15}\right)}^{5.257}\;$$

ZWD varies with time and can be determined by the temperature, pressure, and relative humidity around the GPS station. ZWD can be obtained by subtracting ZHD (Equation (1)) and ZTD from the GPS PPP and is simply expressed as ZWD=ZTD−ZHD. The PWV can be computed by the following:

(4) $$\;\mathrm{PWV}=\frac{10^{5}}{R_{v}\times \left(k_{2}-k_{1}\times \frac{mv}{md}+\frac{k_{3}}{T_{m}}\right)}\cdot \mathrm{ZWD}\;$$

where $R_{v}$ denotes the specific gas constant of water vapour; $k_{1}$, $k_{2}$, and $k_{3}$ are atmospheric refractivity constants; and mv and md are molar mass of water vapour and dry air, respectively. $k_{1}$, $k_{2}$, $k_{3}$ mv and md are $461\;J\cdot {\left(\mathrm{kg}\cdot K\right)}^{-1}$, 77.6 $\;K\cdot {\left(\mathrm{hPa}\right)}^{-1}$, 71.98 $K\cdot {\left(\mathrm{hPa}\right)}^{-1}$, $3.754\times 10^{5}\;K^{2}\cdot {\left(\mathrm{hPa}\right)}^{-1}$, 18.0152 $k\cdot {\left(\mathrm{mol}\right)}^{-1}$, and 28.9644 $k\cdot {\left(\mathrm{mol}\right)}^{-1}$, respectively [30]. $T_{m}$ is the weighted mean temperature in the unit of K near the GPS antenna. $T_{m}$ is integral of vapour pressure and temperature along the zenith direction from GPS antenna geopotential height $z_{0}$ to infinity.

(5) $$\;T_{m}=\frac{\int_{z_{0}}^{+\infty }\frac{e\left(z\right)}{T\left(z\right)}dz}{\int_{z_{0}}^{+\infty }\frac{e\left(z\right)}{T{\left(z\right)}^{2}}dz}\;$$

where e(z) and T(z) are the vapour pressure and temperature at the z geopotential height level, respectively. Bevis gave a practicable model [3] for computing the $T_{m}$. The $T_{m}$ is linearly determined by the temperature above the ground. Other researchers proposed that the weighted mean temperature can be better expressed when the Earth surface vapour pressure is introduced [31,32]. $T_{m}$, which is linearly dependent with Earth’s surface temperature and pressure model, is applied in this study (Equation (6)).

(6) $$\;T_{m}=a_{0}+a_{1}\times \bar{T}+a_{2}\times e_{s}\;$$

where $e_{s}$ is atmospheric pressure at the GPS antenna height. Readings of 84 radiosonde stations distributed over China were applied in estimating the polynomial coefficient $a_{0}$, $a_{1}$, and $a_{2}$, which are 92.61, 0.634, and 0.2797, respectively. The weighted mean temperature $T_{m}$ can be calculated by the readings from this radiosonde sensors using Equation 5. The surface temperature $\bar{T}$ and pressure $e_{s}$ are the readings of radiosonde sensors.

### 2.3. PWV Computed by Radiosonde Balloon Readings

This study also computed the PWV from the radiosonde measurements. Radiosonde balloons are the most popular infrastructure and their precise measurements are normally used by weather forecasting agencies. It is the general approach in understanding the meteorology condition from the ground surface to the atmospheric layer, at which the pressure is 100 hPa. The unit of PWV is metres and can be expressed by the liquid water and water vapour density as follows:

(7) $$\;\mathrm{PWV}=\rho^{-1}\times \int_{0}^{\infty }\rho_{w}dz\;$$

where ρ and $\rho_{w}$ are the liquid water and water vapour density, respectively. Both have a unit of $\mathrm{kg}\cdot m^{-3}$. z is the height and is measured in metres. With the assumption of hydrostatic balance ($dp=-\rho_{d}\cdot g\cdot dz$, $\rho_{d}$ are the corresponding values for dry air), the PWV can be rewritten as follows [33]:

(8) $$\;\mathrm{PWV}=g^{-1}\times \int_{P_{t}}^{P_{s}}rdp=g^{-1}\times \overset{i=m}{\underset{i=1}{\sum }}\left(\left(\frac{r_{i}+r_{i+1}}{2}\right)\times \left(Pres_{i}-Pres_{i+1}\right)\right)\times 0.1\;$$

In Equation (8), g is the Earth gravity accelerator. It is $9.7936\;m\cdot s^{-2}$ in the Zhejiang province. $P_{t}$ and $P_{s}$ are the atmospheric pressure at the ground surface and stratosphere, respectively, and both have units of hPa. m is the number of atmospheric layers at which the radiosonde balloon sensors record the data. $r_{i}$ and $Pres_{i}$ are the water vapour mixing ratio and atmospheric pressure at *i*th layer, respectively. The water vapour mixing ratio has to be derived from the radiosonde readings; Murray (1967) provided the detailed procedures [34].

## 3. Results and Discussions

### 3.1. Comparision between GPS and Radiosonde PWVs

**Figure 3:**
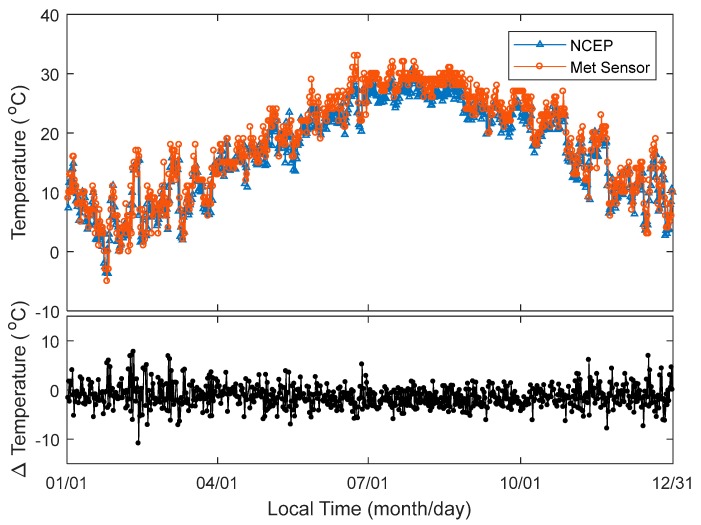
Temperature near CORS Taiz station interpolated by NCEP re-analysed dataset and measured by meteorological sensors at HOJA (top panel) with 12 h intervals over the whole of 2016; and difference of temperature from two approaches (bottom panel) over the whole of 2016.

**Figure 4:**
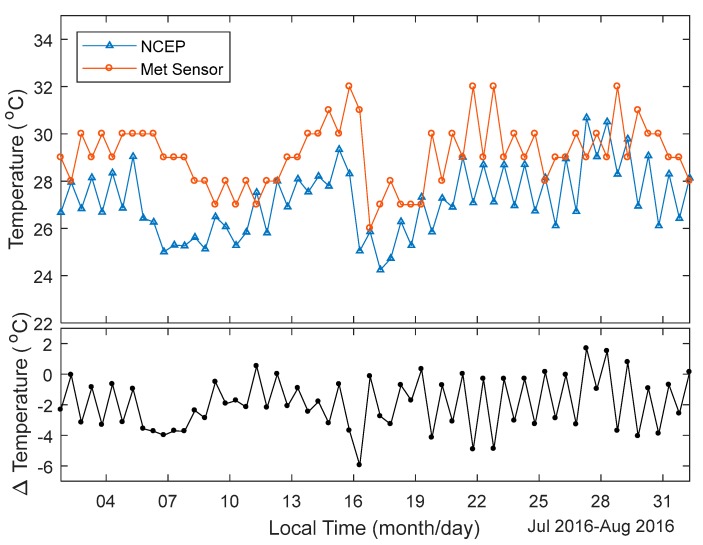
Temperatures at CORS Taiz station and HOJA radiosonde station in July (**top** panel), and the temperature differences in July (**bottom** panel).

Radiosonde meteorological readings were used as a benchmark in comparing the temperature and atmospheric pressure, which is interpolated by the NCEP CFSv2 re-analysed data. The horizontal and vertical distances between GPS antenna and meteorological sensor at the Taiz station are 176.5 m and 16.1 m, respectively. Hence, the meteorological conditions at both places can be treated as the same. Figure 3 top panel shows the temperatures interpolated by the NCEP re-analysed data at the Zhejiang CORS Taiz station. It also shows the temperatures from the radiosonde readings at HOJA, measured twice a day routinely over the whole year of 2016. The trend of temperature changing from NCEP and radiosonde readings are consistent. The bottom panel of Figure 3 shows the time series of temperature difference. Most of the temperature differences are between ±8 °C, only one record exceeds −10 °C. Figure 4 shows the temperatures from the NCEP and radiosonde in July 2016. Most of the interpolated temperatures from NCEP datasets are lower than that from the meteorological sensor readings. The differences in temperature from the interpolated NCEP dataset and meteorological sensor readings are between −6 °C and 2 °C. Relative changes from NCEP CFSv2 interpolated temperature are higher than those from meteorological sensor readings. The temperature does not change as much as shown by the blue line in Figure 4. This implies that the interpolated temperature from NCEP CFSv2 has worse precision than that measured by the temperature sensor.

**Figure 5:**
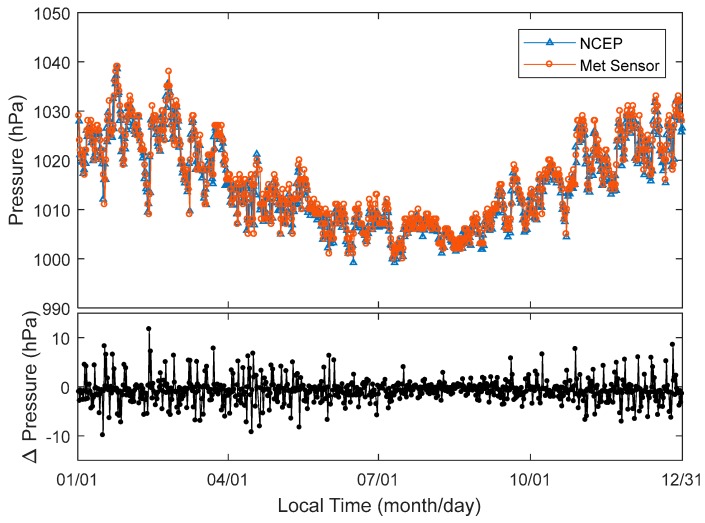
Pressure near GPS antenna measured twice a day and interpolated from NCEP CFSv2 (**top** panel); and difference of pressure from two approaches (**bottom** panel) in the whole year of 2016.

Figure 5 shows the atmospheric pressures interpolated by the NCEP CFSv2 re-analysed data and the radiosonde readings measured twice in a day over the whole of 2016 at Taiz and HOJA, respectively. The trend of atmospheric pressure changing from NCEP and radiosonde agree with each other very well (Figure 5 top panel). However, the differences in atmospheric pressure from NCEP and radiosonde are most evident. The differences in atmospheric pressure from January to the beginning of June and from November to December are higher than those between June and November. Most of the differences in atmospheric pressure are approximately 2 hPa in summer. However, it extends to ±5 hPa in other seasons.

**Figure 6:**
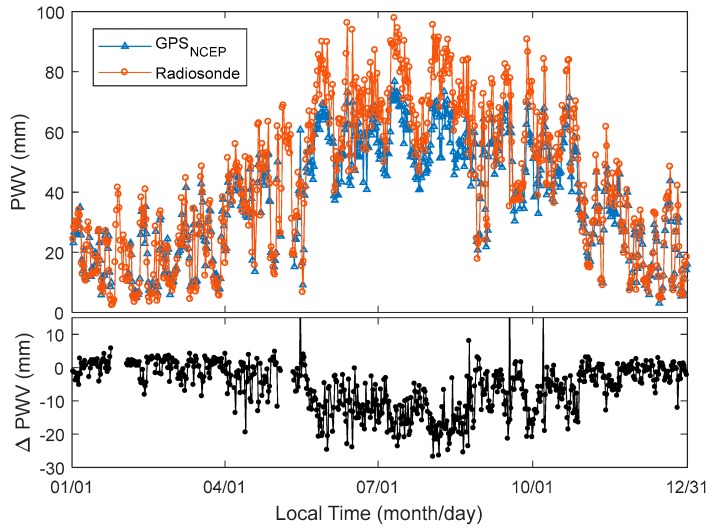
PWV computed by the radiosonde balloon sensors and GPS with NCEP CFSv2 re-analysis dataset (top panel) and PWV differences between radiosonde and GPS and NCEP at Taiz GPS station in 2016 (twice a day, at 07:15 and 19:15).

**Figure 7:**
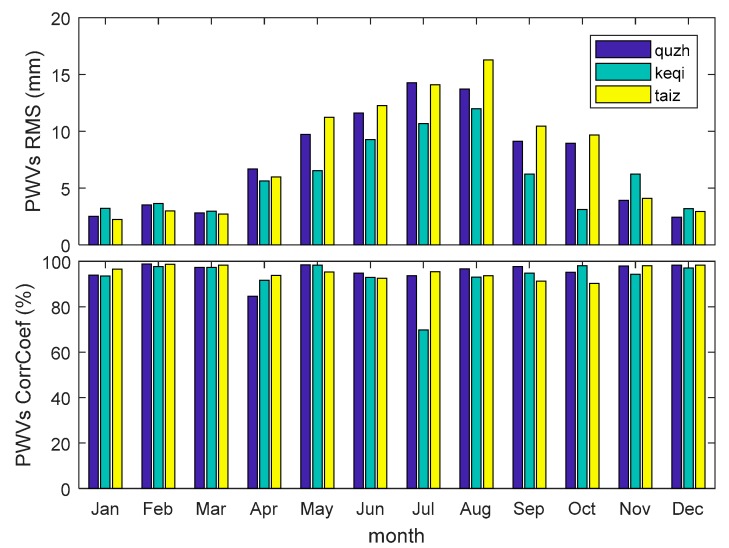
The RMS of GPS PWV and radiosonde PWV differences (top panel) and GPS and radiosonde PWVs correlation coefficient (bottom panel) every month in 2016.

Figure 6 shows the PWV waveforms computed using the radiosonde and data calculated from the GPS PPP and NCEP CFSv2 re-analysed dataset at the station of HOJA and Taiz, respectively. The gradual changes of PWVs from the radiosonde and GPS + NCEP CFSv2 are consistent. This shows the feasibility of using the GPS PPP and NCEP CFSv2 re-analysed dataset for retrieving PWV. The differences of PWVs from radiosonde and GPS are obvious (Figure 6). The PWV from June to October can exceed 20 mm (Figure 6 bottom panel). The RMS of PWV differences between GPS and radiosonde was calculated, which shows that the RMS of PWV differences in January, February, March, November, and December is not more than 5 mm (Figure 7, top panel). It is approximately 5 mm in April. The RMS of PWV differences is bigger and higher, and approaches 10 mm from May to October at Quzh, Keqi, and Taiz. Figure 7 bottom panel shows the correlation coefficient of the radiosonde and GPS PWV time series. Most of the correlation coefficients are more than 90% at the station pairs of Quzh, Taiz, and Keqi. The correlation coefficient of the PWV time series implies that the PWVs estimated from GPS and radiosonde are consistent. By comparing Figure 6 with Figure 3 and Figure 5, the PWV differences of radiosonde and GPS are high between May and October. However, the atmospheric pressure differences occurred between January and the beginning of June, November, and December. This implies that the atmospheric pressure precision does not have a significant effect on the accuracy of PWV retrieved by GPS.

It is necessary to note that the radiosonde balloon records meteorological parameters when it is travelling through the troposphere. This event normally takes a few hours. PWV retrieved by GPS is at the exact epoch when the GPS signal passes through the troposphere. The GPS PPP allows us to obtain the PWV at any time during the extreme weather condition, while radiosonde strictly gives information at 07:15 and 19:15 daily.

**Figure 8:**
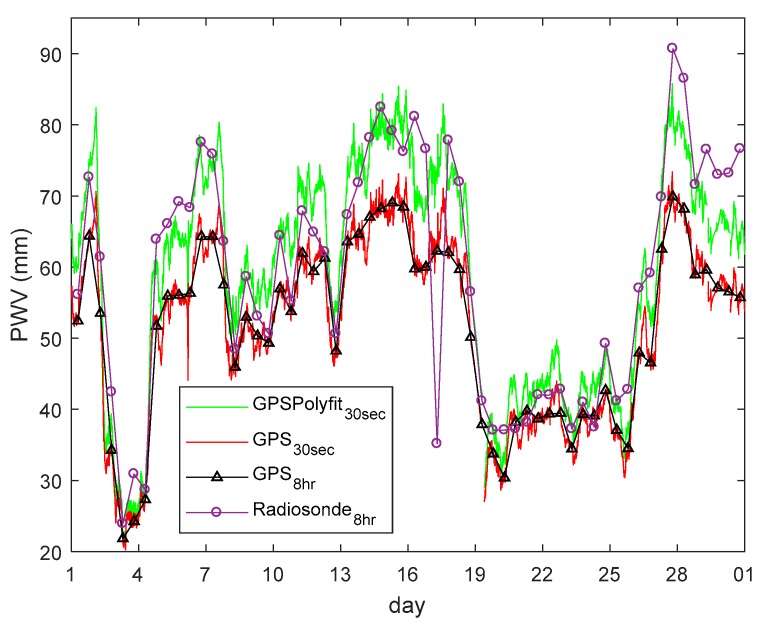
purple line with circle markers and black line with triangle markers represent the radiosonde and GPS PWVs, respectively, every 12 h; and PWV computed by the GPS (red line) and polynomial fit (green line) with a 30 s interval.

The purple line with a circle mark and black line with a triangle mark in Figure 8 are the PWVs in September, which are computed by the radiosonde and GPS, respectively. The PWV from GPS is always lower than that from radiosonde, and the difference can be up to 20 mm (19:15 on 27 September). The red line is the PWV computed by the GPS with a 30 s interval at Taiz station. The Taiz high temporal resolution PWVs and those from other CORS stations allows the analysis of changes in PWV during the typhoon event. However, a bias between GPS and radiosonde PWVs is evident. The polynomial coefficient is computed by the radiosonde PWV at HOJA and GPS PWV at Taiz station. The green line in Figure 8 shows the polynomial fitting of PWV from GPS PWV. GPS PWVs computed from all stations are fitted with a polynomial before being used for understanding the PWV changes during the typhoon event.

### 3.2. PWV Changes over Zhejiang Province during Typhoons

**Figure 9:**
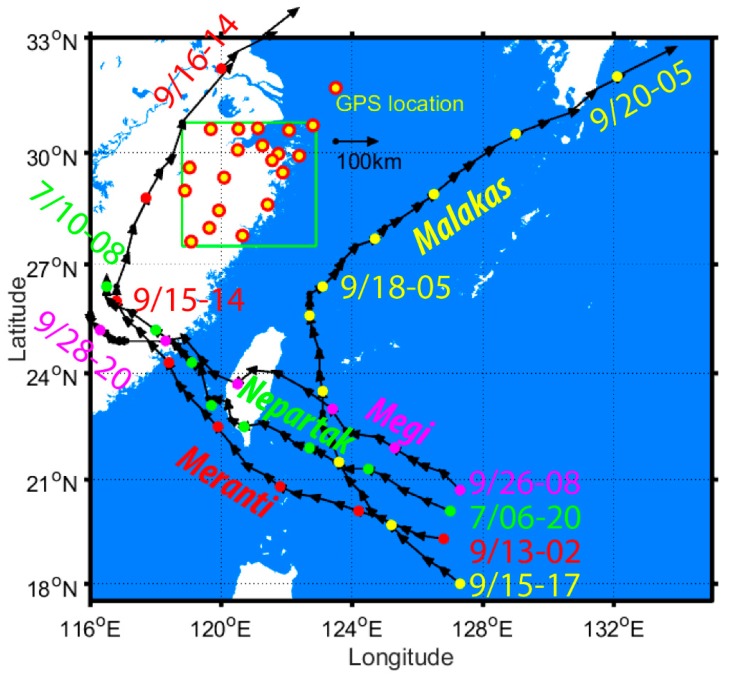
Route of MERANTI, NEPARTAK, MEGI, and MALAKAS typhoons every 3 h from 02:00 on 13 September to 14:00 on 16 September, 20:00 on 6 July to 08:00 on 10 July, 08:00 on 26 on September to 20:00 on 28 on September, and 17:00 on 15 September to 05:00 on 20 September, respectively, in 2016. The period between neighbouring colourful dots on the typhoon path is 12 h.

Typhoons frequently occur around Zhejiang province every year and cause heavy rainfall and flooding. There were four typhoon events that affected the Zhejiang province in 2016. The first one is named “NEPARTAK” and occurred in July 2016. The other three were named “MERANTI”, “MALAKAS”, and “MEGI”, and occurred in September 2016. MERANTI landfall location occurred at Xiamen, Fujian province at 03:05 in September 2016. The wind speed of the typhoon’s centre was up to 48 m/s, with its pressure at 945 hPa. The average rainfall in Ningbo, where University of Nottingham Ningbo China (UNNC) GPS station is located in Figure 9, is 229 mm. The rainfall in some districts in Ningbo was as high as 444 mm during the typhoon event. The NEPARTAK and MEGI typhoons were a little weaker than MERANTI and also caused heavy rainfall in Ningbo. Table 1 gives the wind speed and pressure of the typhoons’ centre, as well as the average and maximum rainfall during the typhoon period. MALAKAS did not arrive in China mainland. The nearest distance between the Ningbo and MALAKAS route is approximate 360 km.

Figure 9 shows the tracks of the NEPARTAK, MERANTI, MALAKAS, and MEGI typhoons in 2016. The green, red, yellow, and magenta dots represent the routes of the NEPARTAK, MERANTI, MALAKAS, and MEGI typhoon centres, respectively. There is a twelve-hour time interval between the two neighbouring dots, during which there are four arrow vectors. Each arrow vector shows the direction and distance of the typhoons’ centre movements over three hours. Each typhoon route also contains the start and end time of the recording in Figure 9. The green box shows the study regions, where 19 CORS stations were selected for calculating PWV. NEPARTAK, MERANTI, and MEGI all made landfall in Fujian province. NEPARTAK and MEGI typhoons became weaker after landfall, and stopped recording their route at 08:00 and 20:00 on 10 July and 28 September, respectively. MERANTI brought heavy rainfall in Ningbo and the whole Zhejiang province. The first section of its route was from southeast to northwest, after which its direction turns to northeast at 14:00 on 15 September. MALAKAS did not make landfall on China mainland from beginning to end. It jointly affected the PWV distribution over Zhejiang province with MERANTI, as these two typhoons overlapped in time. Figure 9 shows MALAKAS first travelling from southeast to northwest, and then in a northerly direction. It changed its direction to northeast at 02:00 on 18 September before leaving Zhejiang province.

**Figure 10:**
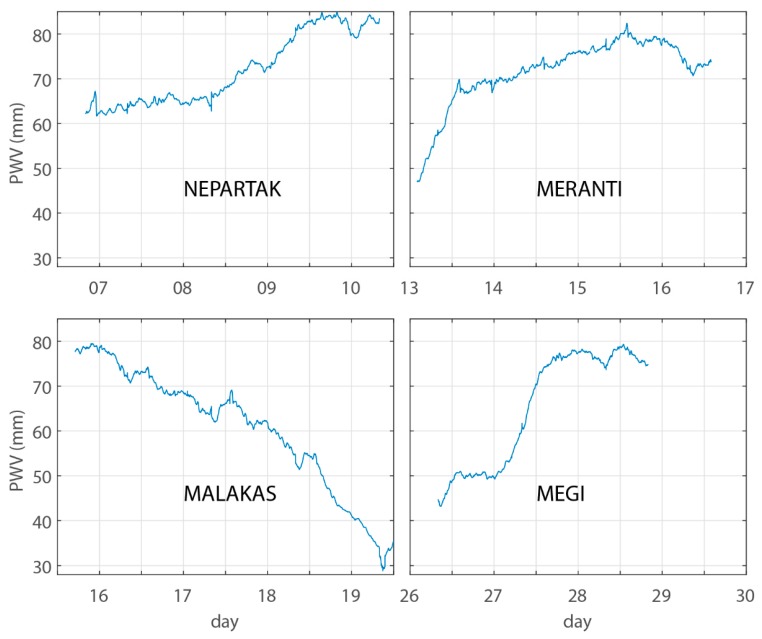
The average PWV over Zhejiang province during NEPARTAK, MERANTI, MALAKAS, and MEGI typhoons. NEPARTAK occurred in July, and the other three typhoons occurred in September.

The PWV calculated from the standalone GPS receiver can only reflect the region’s PWV near the GPS antenna. The PWV was interpolated using 19 CORS stations in Zhejiang province. Figure 10 shows the average PWV over Zhejiang province. The average PWV from three landfall typhoons over China mainland displays an increasing trend. Waveform of average PWV from NEPARTAK is at the normal range of 65 mm, and slightly increased after 8 July, during which the NEPARTAK typhoon centre is approximately located at longitude 123° E and latitude 22.5° N. The average PWV of MERANTI and MEGI typhoons significantly increased after 02:00 on 13 September and 00:00 on 27 September, during which the MERANTI typhoon started to record its route in this study. The centre of MEGI typhoon is located at longitude 124° E and latitude 22.5° N. The significant increase in the average PWV coincides with the heavy rainfall in the province (see Table 1).

**Figure 11:**
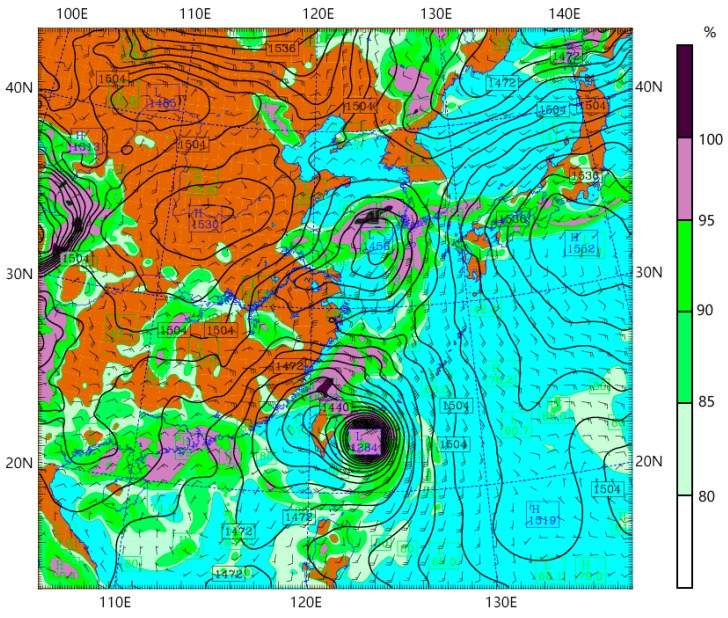
Relative humidity and potential height of the MALAKAS typhoon with the pressure at 850 hPa layer at 02:00 on 17 September.

The bottom left subfigure of Figure 10 shows that average PWV over Zhejiang province kept decreasing during the MALAKAS typhoon period, during which time MALAKAS never made landfall on the China mainland. The severity and compact structure of MALAKAS (see Figure 11) relatively concentrated the vapour around the typhoon centre. The vapour is not advected to Zhejiang province in this case. Figure 11 also shows that there are masses of cold dry air at the northwest of Zhejiang province. This is the major reason that the MALAKAS typhoon changed its direction to northeast at 02:00 on 18 September. The masses of cold dry air decreased the vapour content of air over Zhejiang province, and this agrees with the PWV derived from GPS (Figure 10, bottom left panel).

**Figure 12:**
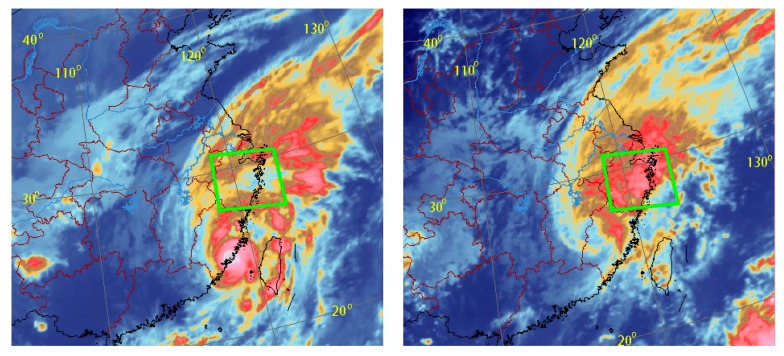
MERANTI satellite images from China FY2E-IR1 satellite at 03:30 (**right** panel) and at 14:30 (**left** panel), respectively, on 15 September. Research areas are highlighted by green boxes (FY2E is one satellite of the Fengyun-2 constellation, IR1 indicates infrared 1 channel).

The route of MERANTI entirely embraces Zhejiang province. Figure 12 shows the infrared satellite images from China FT2E-IR1 at 03:30 and 14:30 on 15 September, at which time the typhoon had just made landfall (left panel) and then began to change direction (right panel) from the northwest to the northeast. The red color in Figure 12 shows convection developing vigorously in that particular region, demonstrated by thick cloud. The green box in Figure 12 shows the research region in this study.

**Figure 13:**
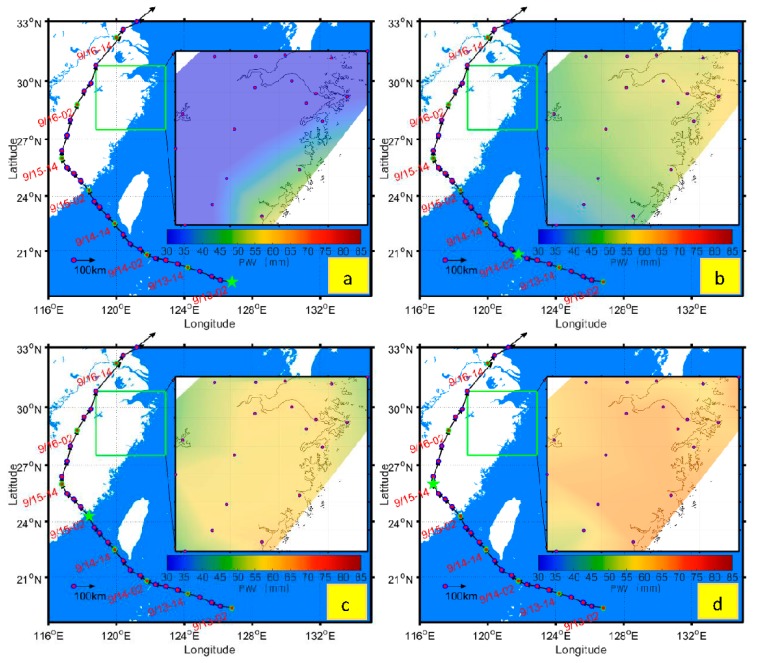

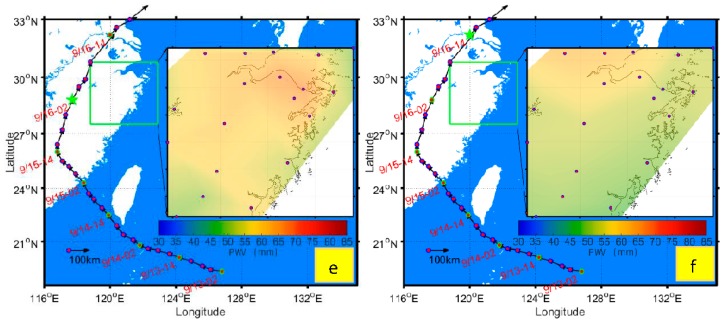
PWV changing before and after the MERANTI landfall. The green five-point-star denotes the location of typhoon epicenter at the particular time in each subfigure marked with (**a**–**f**).

The radiosonde stations launch the air balloons twice a day strictly at 07:15 and 19:15 in China. This means that PWV from radiosonde can only be obtained at specific epochs. It is thus not possible to calculate PWV when there is no radiosonde event or when a typhoon passes a place without a radiosonde station. The PWV over Zhejiang province is plotted twice a day at 02:00 and 14:00 local time. Table 2 shows the minimum, maximum, and average PWV over Zhejiang province during the typhoon event. The average PWV kept increasing when the typhoon travels from southeast to northwest, and decreases when the typhoon changed its direction at 14:00 on 15 September. Figure 13 illustrates the change in trend of PWV according to the typhoon route from 02:00 on 13 to 14:00 on 16 September 2016 over Zhejiang province. Figure 13a shows that PWV over the land near the sea is higher than the inland PWV when the typhoon is far away (Lat: 19.3° N; Long: 126.8° E) from the China mainland. Most of the PWV distributed over Zhejiang province is very low, although it is high at Ruia and Taiz GPS stations. The minimum and maximum PWV are 26.27 mm and 76.13 mm, respectively. Figure 13b,c shows the PWV distributions over Zhejiang province from 02:00 on 14 to 02:00 on 15 September 2016, during which time the typhoon heads to Xiamen Fujian province. The PWV distribution at 14:00 on 13 September and 14:00 on 14 September can be found in Appendix A. Before the typhoon makes landfall on China mainland, the PWV in Zhejiang province gets higher as the typhoon gets closer to China. When MERANTI makes landfall in Xiamen, the PWV in Zhejiang was almost uniformly distributed with a higher PWV (Figure 13c). MERANTI changed its direction to the northeast of China at 14:00 on 15 September, during which time the PWV is highest in Zhejiang province (Figure 13d). The average PWV over Zhejiang province is up to 82.23 mm during this time, and starts to decline when the typhoon travels from southwest to northeast. Figure 13d–f shows that the peak of PWV moved from southwest to northeast of China in same direction to that of the typhoon centre. Figure 13f shows that the PWV distribution over Zhejiang province at which time the MERANTI typhoon is departing. Figure 13d–f reveals that the peaks of PWV distribution are normally in front of the typhoon centre, in the direction of the typhoon movement.

## 4. Conclusions and Recommendations

The temporal–spatial resolution needs to be high in order to study the changing conditions during the typhoon events. This paper took advantage of GPS PPP PWV high temporal–spatial resolution to study the PWV changes during the MERANTI, NEPARTAK, MEGI, and MALAKAS typhoons. The three radiosonde stations in Zhejiang offer the PWV strictly at 07:15 and 19:15. Additionally, the three radiosonde stations are sparsely distributed in Zhejiang province (105,500 ${\mathrm{km}}^{2}$). Thus, the temporal–spatial resolution cannot achieve the requirement in understating PWV changes. GPS PWV retrieval can significantly enhance the temporal resolution. The NCEP CFSv2 re-analysed dataset was used for interpolating the temperature and atmospheric pressure near the GPS antenna because the GPS station is not equipped with meteorological sensors. The measured temperature and pressure were used as a benchmark in comparison with the interpolated NCEP dataset. The maximum temperature and pressure differences between measured and interpolated are 6 degrees and 2 hPa, respectively. A procedure of computing PWV by radiosonde raw readings is described in this paper. One-year PWV at GPS Taiz station has been calculated by the GPS and NCEP re-analysed data and radiosonde readings. The results show that GPS and radiosonde PWV biases are very small in January, February, March, November, October, and December, with their RMS not more than 5 mm. The RMS bias of PWV in April is approximately 5 mm. The PWV biases in May, June, July, August, September, and October are evident with RMS up to 10 mm and more than 15 mm in August at the Taiz station. This is because the weighted mean temperatures during these months have bias using the model (Equation (6)), the polynomial coefficients of which were estimated over the entire year. Polynomial coefficients of Equation (6) are recommended to be estimated monthly for avoiding GPS and radiosonde PWV biases. The correlation coefficient of GPS and radiosonde PWV differences implies that the changes in PWVs are consistent. The PWV retrieved using GPS PPP and NCEP CFSv2 re-analysed datasets is credible. The polynomial coefficient is estimated by the PWV from the Taiz GPS station and HOJA radiosonde PWV. The estimations were then applied to the other PWV CORS stations. The results of the 19 GPS stations were used for understanding the PWV changing characteristics of the four typhoons. The three landfall typhoon results shown that average PWV over Zhejiang province kept increasing when the typhoon approached China mainland. MALAKAS did not make landfall in China mainland. Its compact structure concentrated the vapour near the typhoon center, which prevents vapour from being translated to Zhejiang province. Masses of cold dry air with high pressure from northwest of Zhejiang province prevented the MALAKAS route from extending to China mainland and changed its direction from northwest to northeast. The cold dry air from northwest of Zhejiang province was one of the reasons that the PWV decreased. This is consistent with the GPS PWV during MALAKAS. PWV spatial distribution on the arrival of MERANTI shows that the PWV over the coast is larger than those inland before and after the typhoon. The results also show that the peak of PWV over Zhejiang province follows the route of the MERANTI typhoon’s epicentre and is always ahead of the typhoon’s epicentre.

## Figures and Tables

**Table 1:** NEPARTAK, MERANTI, and MEGI typhoons wind speed and pressure during landfall, and the average/maximum rainfall during the typhoon periods.

	Landfall		Rainfall in Ningbo	
	Wind Speed (m/s)	Pressure (hPa)	Average (mm)	Maximum (mm)
NEPARTAK	28	985	26.5	93
MERANTI	48	945	229	444
MEGI	33	975	83.4	236.2

**Table 2:** The minimum, maximum, and average precipitable water vapour (PWV) over Zhejiang province every 12 h from 02:00 on 13 September to 14:00 on 16 September 2016.

Time	09/13 02:00	09/13 14:00	09/14 02:00	09/14 14:00	09/15 02:00	09/15 14:00	09/16 02:00	09/16 14:00
Min. (mm)	26.27	45.14	55.84	64.44	65.83	72.20	68.89	66.94
Max. (mm)	76.13	81.47	78.76	82.73	81.28	86.07	86.88	82.21
Avg. (mm)	46.91	69.84	70.10	74.82	75.80	82.23	77.65	73.67

**Figure A1:**
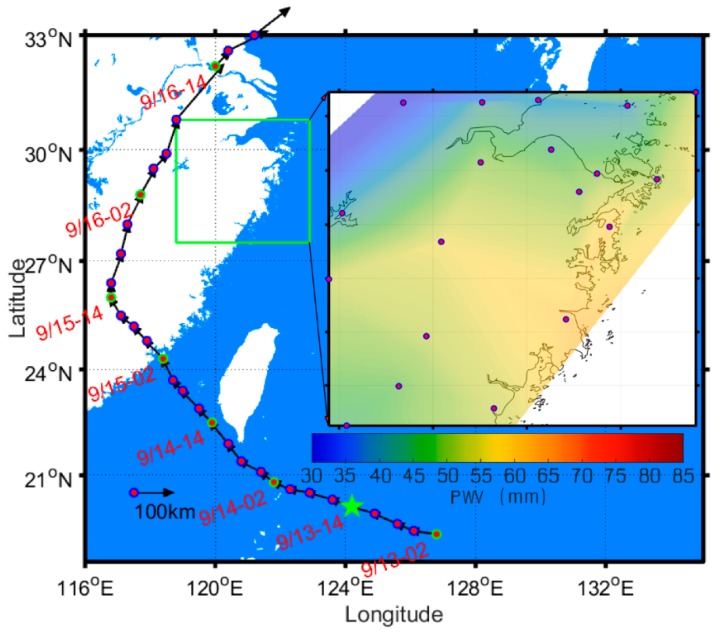
The PWV distribution over Zhejiang province at 14:00 on 13 September 2016.

**Figure A2:**
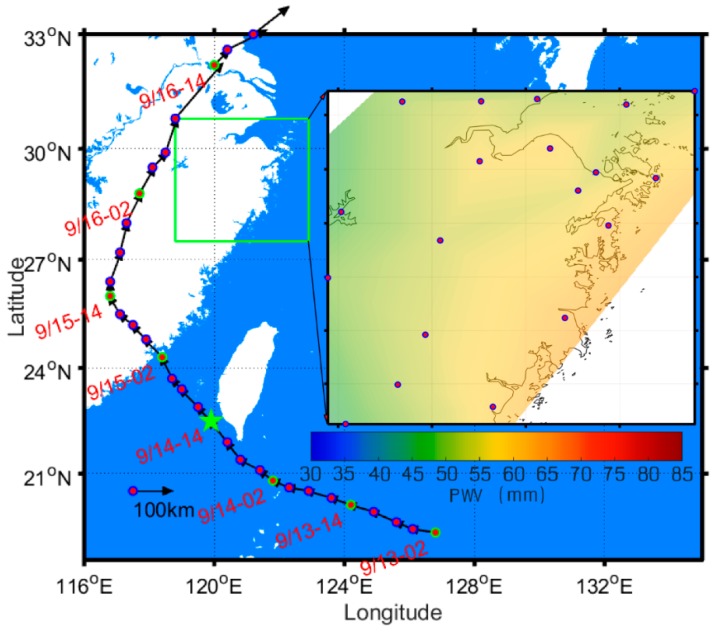
PWV distribution in Zhejiang province at 14:00 on 14 September 2016.

**Figure A3:**
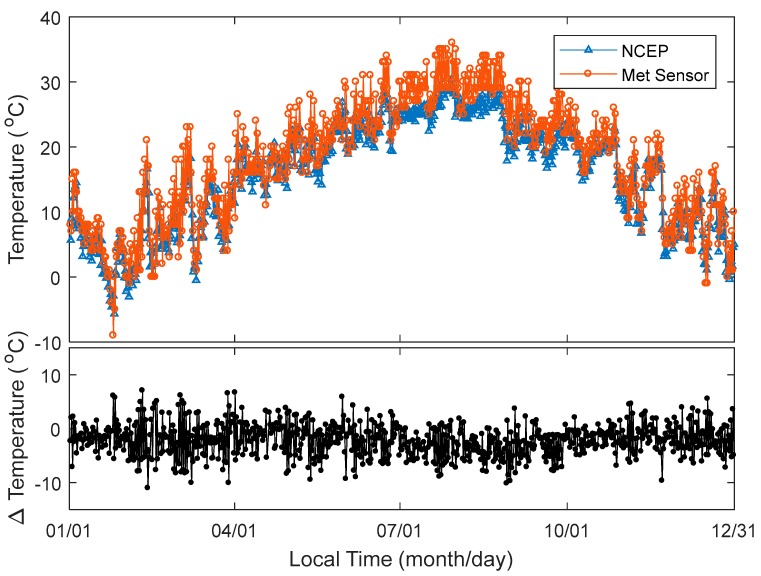
The temperature time series from NCEP and radiosonde at GPS Quzh station and QUZO radiosonde station, respectively.

**Figure A4:**
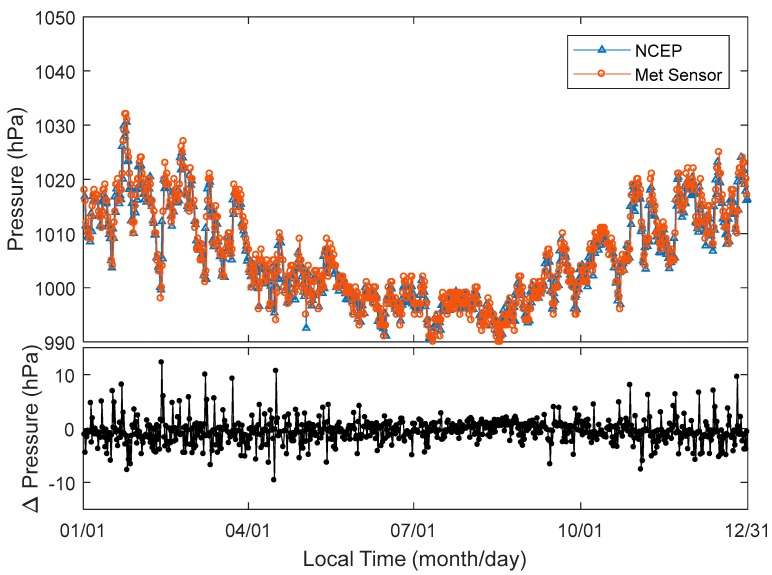
The atmospheric pressure time series from NCEP and radiosonde at GPS Quzh station and QUZO radiosonde station, respectively.

**Figure A5:**
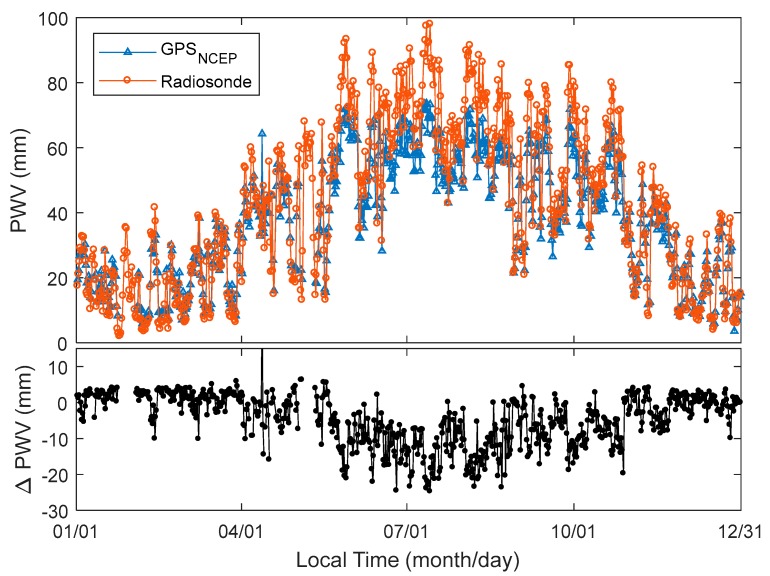
The PWV time series from GPS and radiosonde at GPS Quzh station and QUZO radiosonde station, respectively.

**Figure A6:**
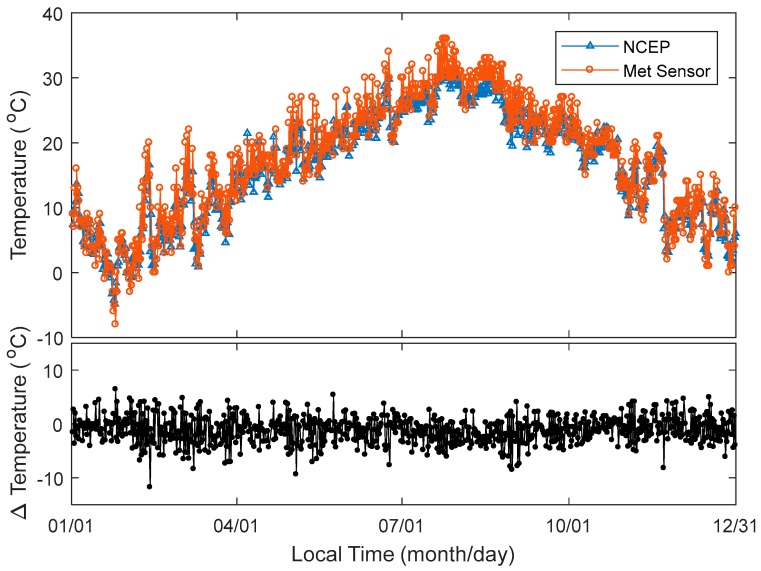
The temperature time series from NCEP and radiosonde at GPS Keqi station and HAZH radiosonde station, respectively.

**Figure A7:**
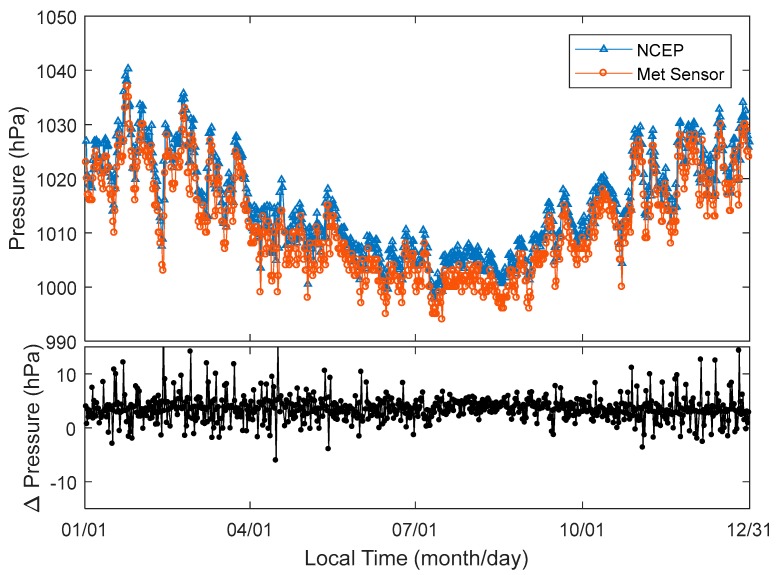
The atmospheric pressure time series from NCEP and radiosonde at GPS Keqi station and HAZH radiosonde station, respectively.

**Figure A8:**
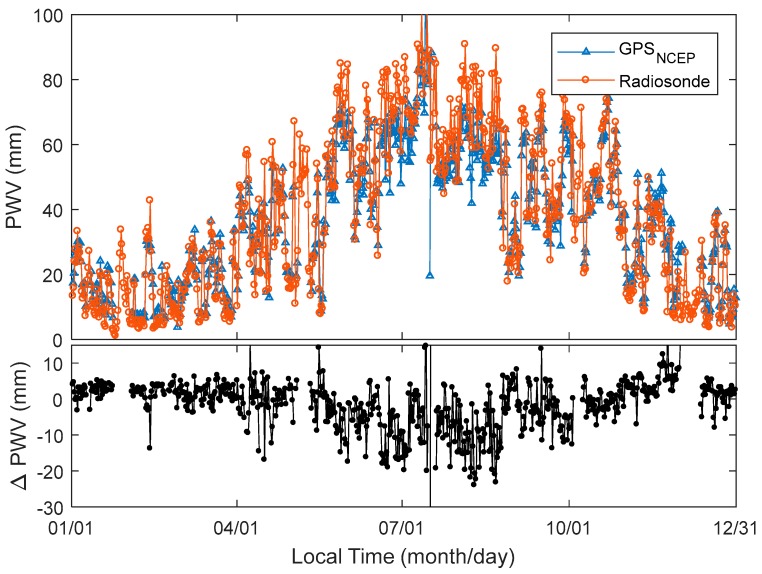
The PWV time series from GPS and radiosonde at GPS Keqi station and HAZH radiosonde station, respectively.
